# Affinity improvement of specific scFvs interacting with the *Neisseria meningitidis* factor H-binding protein: a molecular dynamics simulation approach and experimental study

**DOI:** 10.1186/s12896-026-01204-3

**Published:** 2026-07-25

**Authors:** Bijan Ghazanfari, Zahra Ebrahimi, Fatemeh Yarian, Arezu Lari, Ali Ghanbariasad, Bahram Kazemi

**Affiliations:** 1 Department of Medical Biotechnology, School of Advanced Technologies in Medicine, Fasa University of Medical Sciences, Fasa, Iran; 2 Department of Medical Biotechnology, School of Advanced Technologies in Medicine, Shahid Beheshti University of Medical Sciences, Tehran, Iran; 3 Ludwig Boltzmann Institute of Osteology, Vienna, Austria; 4 Cellular & Molecular Biology Research Center, Shahid Beheshti University of Medical Sciences, Tehran, Iran

**Keywords:** *N. meningitidis*, Affinity improvement, Factor H binding protein, Single-chain variable fragment, Molecular dynamics simulation

## Abstract

**Background:**

In silico approaches are becoming increasingly important for enhancing the affinity of antibodies and antibody fragments, including single-chain variable fragments (scFvs). In this study, we present an in silico affinity-maturation workflow designed to improve the affinity of an scFv directed against *Neisseria meningitidis* factor H-binding protein (fHbp) by integrating in silico maturation, molecular dynamics (MD) simulations, and experimental validation. Approximately 300 single-point substitutions were designed at scFv residues predicted to interact with fHbp, and the resulting V3 (R99D) scFv–fHbp complexes were analyzed through all-atom MD simulations. Guided by bioinformatic analyses, the top V3 (R99D) variant was cloned and expressed, and its binding activity was validated by ELISA.

**Results:**

MD analyses identified five variants, Y35H, L31R, R99D, R99H, and A100K, that exhibited markedly enhanced complex stability. In all variants, the scFv secondary structure remained intact, whereas fHbp showed shrinkage relative to the scFv in the V3 (R99D)–fHbp complexes during the simulations. Hydrogen-bonding patterns and inter-residue distances further supported the improved affinity of the variants relative to the native form. The R99D variant showed the highest binding affinity, with an experimentally measured value of 67.93 × 10⁹ M⁻¹.

**Conclusion:**

Collectively, these findings underscore affinity maturation as a powerful strategy for engineering high-affinity antibody fragments.

**Supplementary Information:**

The online version contains supplementary material available at https://doi.org/10.1186/s12896-026-01204-3.

## Background

Rational improvement of the binding affinity of protein–protein interactions (PPIs), including antigen–antibody interactions, remains a major goal in protein engineering and in the development of high-affinity antibodies for the diagnosis and treatment of various diseases. Accordingly, many studies have focused on enhancing PPIs through random or site-directed mutagenesis [1–3]. Rational improvement of the binding affinity of protein–protein interactions (PPIs), including antigen–antibody interactions, remains a major goal in protein engineering and in the development of high-affinity antibodies for the diagnosis and treatment of various diseases. Accordingly, many studies have focused on enhancing PPIs through random or site-directed mutagenesis [4].

Advances in antibody technology, together with growing knowledge of antibody–target interactions, have made it possible to design and optimize engineered antibodies more effectively. The physicochemical properties of specific residues, along with energy scores, are important criteria in antibody engineering. Over the past decade, several computer-based design strategies have been developed to improve antibody affinity with far greater speed and accuracy [5]. These approaches may provide novel routes for designing therapeutic antibodies against numerous pathogens that have resisted traditional vaccine development, including bacteria and highly antigenically variable viruses such as HIV, HCV, influenza, and Ebola virus [6].

Factor H-binding protein (fHbp), also known as GNA1870, plays an important role in the pathogenesis of *Neisseria meningitidis* and in vaccine design [7]. Because it mediates primary interaction with human factor H (fH), thereby downregulating the alternative complement pathway and facilitating innate immune evasion, fHbp is essential for the survival of *N. meningitidis* in the human host [8]. It is therefore a valuable therapeutic target for bactericidal antibodies. Since the interaction with human factor H occurs on an extracellular and structurally accessible surface, fHbp represents an attractive target for antibody-based recognition and engineering. Consequently, a detailed characterization of the physicochemical determinants of this protein–protein recognition, including interface residues, hydrogen bonding, steric complementarity, and electrostatic interactions, is essential for the rational design of higher-affinity binders.

In our previous study, a novel single-chain variable fragment (scFv) antibody against *N. meningitidis* fHbp was selected and developed by ribosome display [9–11]. In the present study, we employed a structure-based computational design strategy combined with a ligand-based affinity-improvement approach to enhance the affinity of the anti-fHbp scFv. To this end, molecular docking was used to identify key scFv residues involved in antigen–scFv interactions. To design and subsequently select the best mutants, single-point substitutions and descriptor-based screening were applied. Using all-atom MD simulation and the umbrella sampling method, the binding free energy of five variants and the extent of affinity improvement were calculated. The selected antibody was recombinantly produced, and its binding affinity was confirmed in vitro.

## Methods

### Model generation and docking

Given the pivotal role of complementarity-determining regions (CDRs) in mediating antigen recognition and binding in antibody structures, their precise identification and characterization formed the foundation of the present investigation. Defining these hypervariable loops in the antibody framework, together with their specific contributions to the initial antigen–antibody complex, represented the first essential step in this study. The parental scFv sequence used here is publicly available in GenBank under accession number LC191294. Accordingly, the PIGS server (https://bio.tools/pigs) was used to generate a three-dimensional wild-type scFv model [10, 12]. PIGSpro is a robust, biophysically grounded antibody-structure prediction platform that uses canonical conformations to model CDR loops with high precision. By integrating an updated template library, refined predictions of VH–VL domain orientation, and improved handling of non-canonical conformations, it remains a reliable benchmark in the era of rapidly advancing deep-learning methodologies. Its transparency and reproducibility make it valuable for therapeutic antibody design, humanization, and paratope optimization, while its open-access format supports broad use in academic research. For the antigen model, the fHbp crystal structure (PDB code: 2KC0, 2.86 Å) was used. Critical residues involved in the interaction between scFv and fHbp were identified by docking with the ClusPro server (https://cluspro.bu.edu) [13].

Following this, a structure-guided mutation-design strategy was applied to select candidate mutations. Approximately 300 single-point mutants were evaluated according to several criteria: (i) spatial proximity to the interface, (ii) potential for hydrogen-bond formation, (iii) improved electrostatic complementarity, and (iv) steric compatibility. The resulting three-dimensional models of the variants were docked with fHbp. Altogether, a comprehensive set of 300 molecular docking runs was conducted to systematically explore the binding interactions. The docking energy scores were used for primary screening. This process led to the prioritization of five variants, Y35H, L31R, R99D, R99H, and A100K, for detailed MD simulation and experimental validation. A concise summary of the mutation-design workflow, screening criteria, and progression from approximately 300 single-point mutants to the five selected variants is provided in Supplementary Table S1.

The selection of these variants was driven by computational analysis of the scFv–fHbp interface. In particular, R99D was identified as a promising candidate because it could introduce a beneficial charge reversal at the binding site, thereby enhancing electrostatic complementarity with the antigen. The selected variants were subsequently evaluated in terms of root mean square deviation (RMSD), radius of gyration (Rg), and root mean square fluctuation (RMSF). All visualizations were generated using Chimera. In addition, the LIGPLOT program was used to identify residues involved in ligand interactions [14].

### MD simulations

All-atom molecular dynamics (MD) simulations were performed using the GROMACS software package (version 4.5.3) and the GROMOS96 43a1 force field [15] to systematically investigate the structural dynamics, conformational stability, and key intermolecular interactions between the engineered single-chain variable fragment (scFv) mutants and factor H-binding protein (fHbp) within the resulting antigen–antibody complexes. The GROMOS96 43a1 force field was selected because of its well-documented reliability in simulating protein–protein interfaces, particularly in systems involving flexible loops, side-chain reorientation, and long-range electrostatic contributions that are critical for binding [15].

In addition to geometric complementarity, the evaluation of these variants explicitly considered electrostatic compatibility, because charged substitutions at the binding interface were expected to exert a substantial influence on association affinity. To realistically represent the solvent environment, the simple point-charge (SPC) water model was used to explicitly model water molecules [16]. This three-site rigid water model offers a favorable balance between computational efficiency and accuracy in reproducing the key thermodynamic and structural properties of bulk water under ambient and physiological conditions. The initial configurations were obtained directly from the Protein Data Bank (PDB) structures of the native scFv–fHbp complex, as well as from each mutant scFv complexed with fHbp. Prior to simulation, these structures underwent standard preparation steps, including the addition of missing hydrogen atoms, assignment of appropriate protonation states at neutral pH, and verification of bond lengths and stereochemistry. Each complex was then centered in a rhombic dodecahedral periodic simulation box with a minimum distance of 2.0 nm between any protein atom and the box boundary, thereby ensuring sufficient solvent padding to minimize periodic boundary artifacts and permit full conformational sampling over the course of the trajectory. The box was subsequently filled with pre-equilibrated SPC water molecules [16], with any waters overlapping protein atoms removed. The net charge of each solvated system was neutralized by adding the appropriate number of Na⁺ and Cl⁻ counterions. To further mimic physiological conditions, additional Na⁺ and Cl⁻ ions were introduced to achieve a final ionic strength of 150 mM, which is representative of the ionic environment typically encountered in serum or extracellular fluid, where antibody–antigen recognition occurs in vivo. Proper ionic strength is essential for accurately capturing electrostatic screening, solvation effects, and the overall stability of the protein complex during the MD trajectory.

### Expression of the V3 (R99D) scFv in *E. coli*

The mutant scFv gene (V3) was synthesized into the pET28a plasmid (Bioneer, Korea), and the expression vector was subsequently transformed into *Escherichia coli* BL21 (DE3). The amino acid sequence of the V3 (R99D) scFv is provided in Supplementary File S1.

Single colonies were cultured in 50 mL of LB broth containing 30 µg/mL kanamycin at 37 °C and 200 rpm until the OD600 reached 0.8. Recombinant protein expression was then induced with 1 mM IPTG (Merck, Germany). The His-tagged protein was analyzed by SDS–PAGE on 12% polyacrylamide gels, followed by Coomassie Brilliant Blue R-250 staining. For Western blotting, the protein was transferred to nitrocellulose membranes (Whatman, UK) and probed with an HRP-labeled anti-His-tag monoclonal antibody (Abcam, UK) at a 1:1000 dilution. Immunoreactivity was visualized using DAB/H₂O₂ substrate solution (Roche, Germany).

### Purification of the V3 (R99D) scFv

The recombinant V3 (R99D) scFv was purified using Ni²⁺-NTA agarose resin according to the kit protocol (Novagen, USA). First, the cell pellets were suspended in 5 mL of denaturing buffer (6 M urea, 20 mM NaH₂PO₄, and 500 mM NaCl, pH 8.0), lysed by sonication on ice, and centrifuged at 12,000 × g for 20 min. The resulting supernatant was then loaded onto the resin, washed three times with 15 mL of wash buffer containing an imidazole gradient (10, 20, and 30 mM), and eluted with 1 mL of elution buffer containing 500 mM imidazole. Finally, the eluted protein was dialyzed against PBS at room temperature for five hours.

### Affinity analysis of selected scFv

The affinity constant (Kaff) of the selected scFv for the recombinant antigen fHbp was determined as described by Beatty et al. [17]. An ELISA microplate was coated overnight at 4 °C with S-tagged fHbp antigen at concentrations of 2, 1, 0.5, and 0.25 µg/mL in PBS (pH 7.2). Purified scFv antibodies at concentrations of 5, 2.5, 1.25, and 0.625 µg/mL were applied to the corresponding antigen concentrations. All ELISA experiments were performed with three independent biological replicates, defined as separate protein expression and purification batches followed by independent ELISA runs, with each condition tested in technical triplicate. Next, a horseradish peroxidase (HRP)-conjugated anti-His-tag antibody (Abcam, UK) at a 1:10,000 dilution was used as the secondary antibody. The mixture was incubated for 1 h at room temperature. The HRP substrate 3,3′,5,5′-tetramethylbenzidine (TMB) was then added, and the reaction was allowed to proceed for 10 min. The reaction was terminated by adding 50 µL of 2 N H₂SO₄, and the absorbance was measured at 450 nm. Data are presented as the mean ± standard deviation (SD) of the biological replicates. The apparent affinity constant (Kaff) was calculated using the Beatty equation: Kaff = (*n* − 1)/2(n [Ab′] − [Ab]), where n = [Ag]/[Ag′]. No additional statistical tests for significance between the native and V3 (R99D) scFv were performed in this study, because the primary aim was to quantify the affinity improvement of the R99D variant relative to the previously reported native scFv value [9].

## Results

The initial molecular docking analysis of the interaction between the native antibody and the antigen identified the key amino acid residues of the antibody that contribute to antigen binding. Table 1 lists the residues involved in scFv CDR binding to fHbp through hydrogen-bonding and hydrophobic interactions. Based on this interaction profile, single-point mutations were introduced at selected positions to evaluate their individual contributions to binding affinity. The mutations were designed either to strengthen or to disrupt specific interface features, including hydrogen bonding, electrostatic interactions, and steric fit. After evaluating approximately 300 docking outcomes, five scFv variants were selected for detailed analysis: Variant 0, native (wild-type) scFv, used as the reference control; Variant 1 (Y35H), a tyrosine-to-histidine substitution designed to assess changes in aromaticity and hydrogen-bonding potential; Variant 2 (L31R), a leucine-to-arginine substitution used to assess the effect of introducing a positive charge at the interface in CDR1-VH; Variant 3 (R99D), an arginine-to-aspartate substitution in FR3-VH intended to reverse the charge and probe the role of ionic interactions; Variant 4 (R99H), an arginine-to-histidine substitution at the same position to preserve charge potential while introducing imidazole functionality; and Variant 5 (A100K), an alanine-to-lysine substitution in CDR3-VH designed to introduce a flexible positive side chain and explore extended electrostatic reach. All variants, together with the native control, underwent all-atom molecular dynamics simulations under identical conditions to compare conformational behavior, interface stability, and persistent interaction patterns relative to the wild-type complex. Notably, all selected variants were located in the antibody VH chain.

**Table 1:** The residues involved in the binding of scFv to fHbp

scFv	fHbp
Tyr140	Leu107, leu108
Thr136	Gln106
Tyr135	Gln106
Ser139	Ser139
Tyr141	His138
Asn160	Glu137
Asn163	Glu137
Tyr94	His138
Ala208	Gln134
Arg207	Gly140
Tyr209	Thr109
Glu93	Lys141
Asn92	Lys141
His91	Met142
Ser50	Met142
Ser52	Gln132
Tyr32	Val143, val133
Lys31	Glu131
Ser30	Arg146
Ser28	Arg146

### Energy computation of the model

Table 2 presents the ClusPro energy scores for the native form and the best-designed mutants. The ClusPro energy score represents the interaction energy between two proteins and is expressed as E = Erep + Eattr + Eelec + EDARS, where these terms correspond to the repulsive and attractive components of van der Waals interactions, electrostatic energy, and DARS energy, respectively. The DARS term is a pairwise, structure-based potential constructed from decoys as the reference state.

In this study, the total energy was calculated using weighted coefficients as follows:

$$\:\mathrm{E}\hspace{0.17em}=\hspace{0.17em}0.40\:{\mathrm{E}}_{\mathrm{r}\mathrm{e}\mathrm{p}}\:+\:0.40\:{\mathrm{E}}_{\mathrm{a}\mathrm{t}\mathrm{t}}\:+\:600\:{\mathrm{E}}_{\mathrm{e}\mathrm{l}\mathrm{e}}\:+\:1.00\:{\mathrm{E}}_{\mathrm{D}\mathrm{A}\mathrm{R}\mathrm{S}}$$

**Table 2:** Cluspro energy of native scFv-fHbp and the five variants

Mutants	CDR or FR	Cluspro energy
Native scFv-fHbp		-352.9
V1 mutant scFv-fHbp	CDR1 (VH)	-413.3
V2 mutant scFv-fHbp	CDR1 (VH)	-425.6
V3 mutant scFv-fHbp	FR3 (VH)	-430.4
V4 mutant scFv-fHbp	FR3 (VH)	-410.2
V5 mutant scFv-fHbp	CDR3 (VH)	-436.6

### MD properties of the complexes

**Fig. 1:**
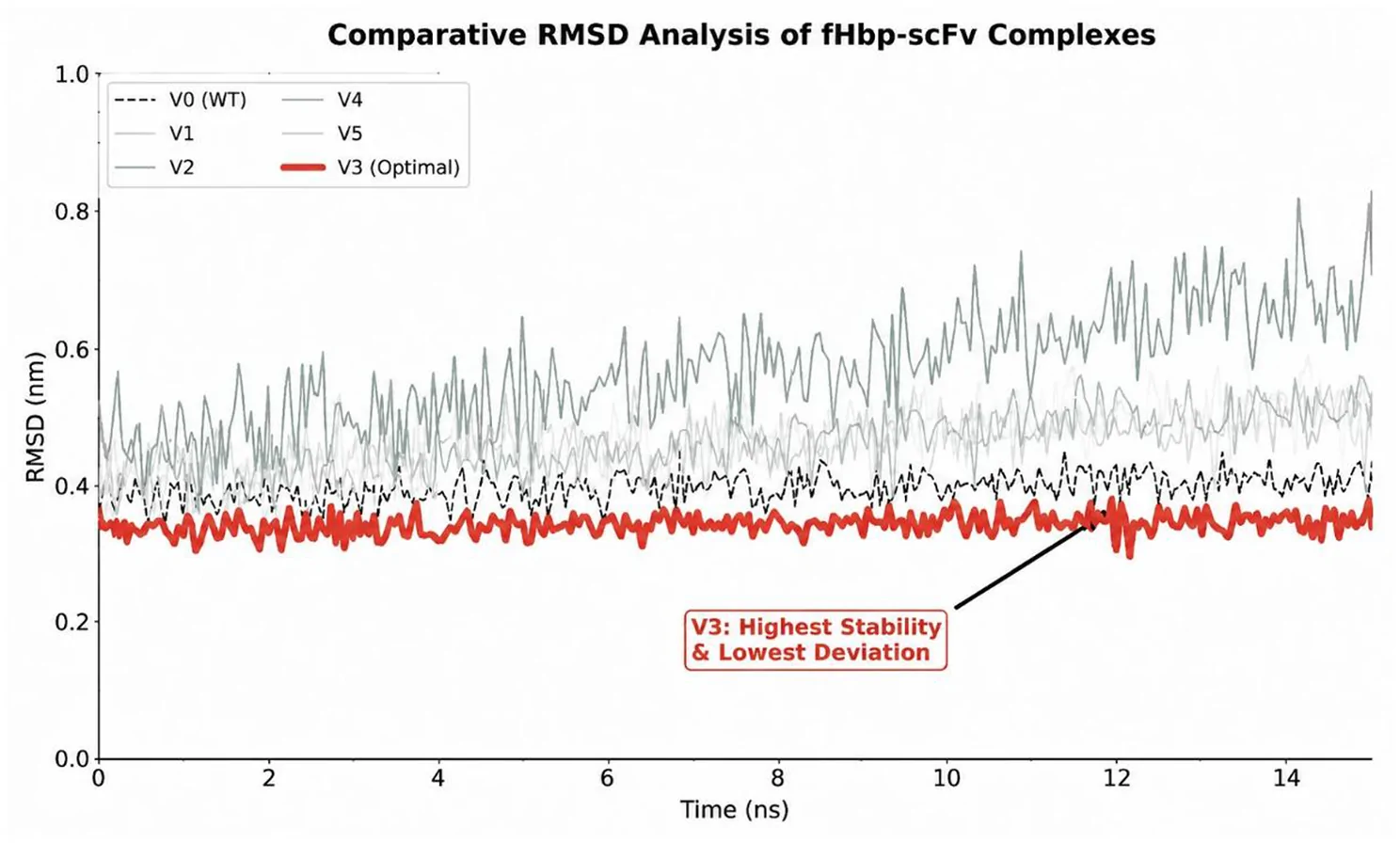
Comparative RMSD analysis of scFv-fHbp complexes among wild-type (V0) and mutant variants (V1–V5) during 15 ns molecular dynamics simulations. The trajectories demonstrate the relative backbone stability of each variant complex over time. Among all mutants, V3 exhibits the lowest and most stable RMSD fluctuations, indicating superior structural stability and conformational convergence compared to other variants. Lighter tones represent less stable or higher deviation variants, while the thicker red trace highlights V3, visually emphasizing its improved stability profile

For clarity, V0 denotes the native scFv, whereas V1 through V5 denote Y35H, L31R, R99D, R99H, and A100K, respectively. To assess stability during the simulations, RMSD, Rg, and RMSF were calculated for fHbp, the scFv, the mutant variants, and their complexes. Figure 1 shows the RMSD values of the variants over time; faster stabilization and lower oscillation indicate more rapid attainment of structural stability. Stable, low RMSD profiles across all components indicate preserved structural integrity and limited unfolding or dissociation over the simulation timescale for all variants. V3 exhibited the lowest RMSD fluctuation, suggesting superior structural consistency within the fHbp–scFv complex, and reached a plateau after 9 ns. In contrast, V1 and V4 showed the largest RMSD fluctuations, indicating that their mutations (Y35H and R99H, respectively) were less stable and required more time to equilibrate.

**Fig. 2:**
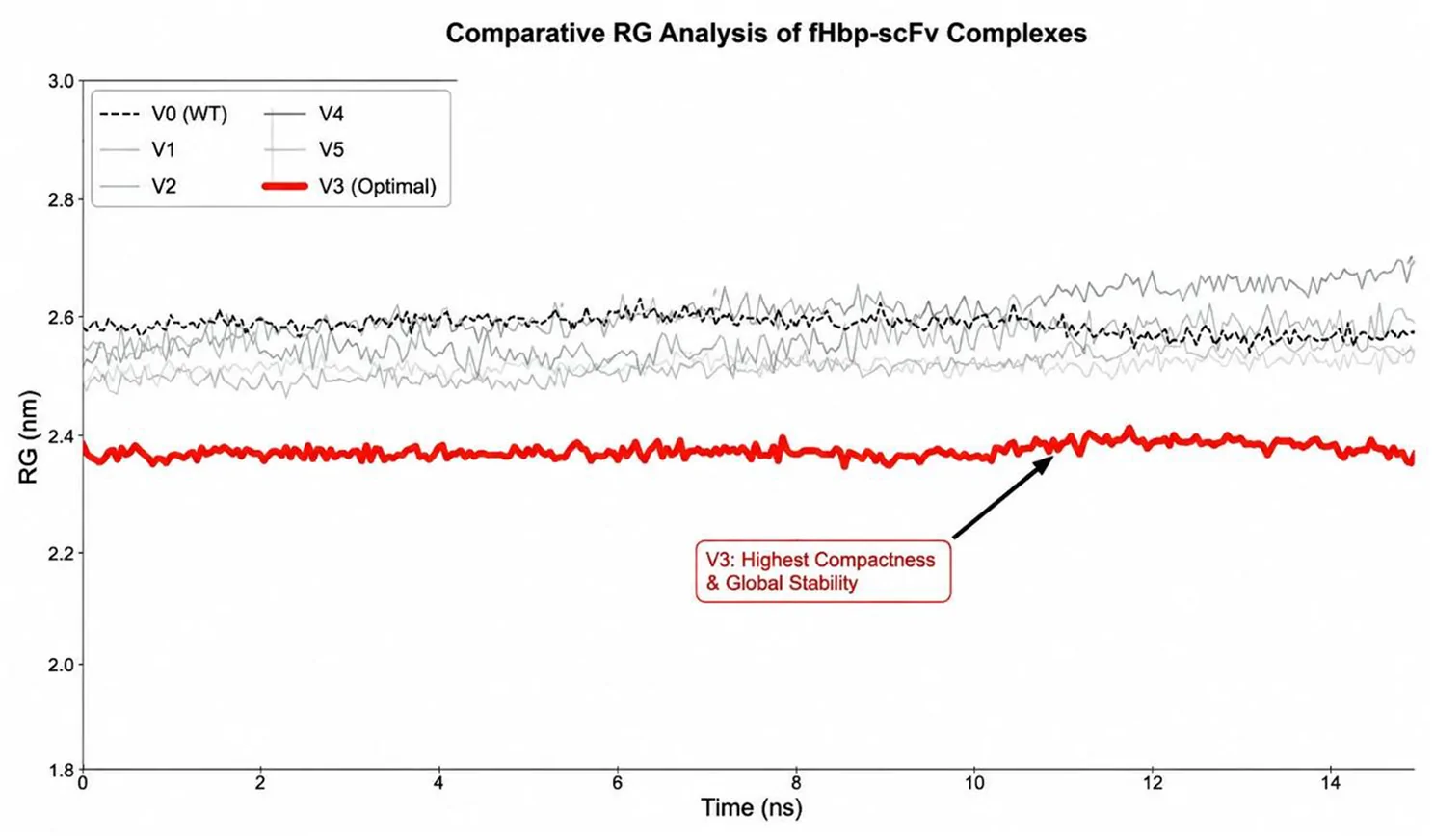
Radius of gyration (Rg) of native (V0) and engineered scFv–fHbp complexes during 15 ns of molecular dynamics simulation. V3 (R99D) showed the lowest Rg fluctuation, indicating a comparatively compact and stable complex

Rg analysis was used to further examine complex unfolding. An increase in Rg may indicate unfolding or folding events. Figure 2 shows that all variants displayed low Rg fluctuations, suggesting high stability. Relatively flat and stable Rg profiles across all components and variants indicate preservation of compact globular structures, with no major unfolding or expansion observed over the simulation timescale. Notably, V3 exhibited the lowest Rg fluctuation when fHbp was complexed with the scFv.

### Secondary structure changes

**Fig. 3:**
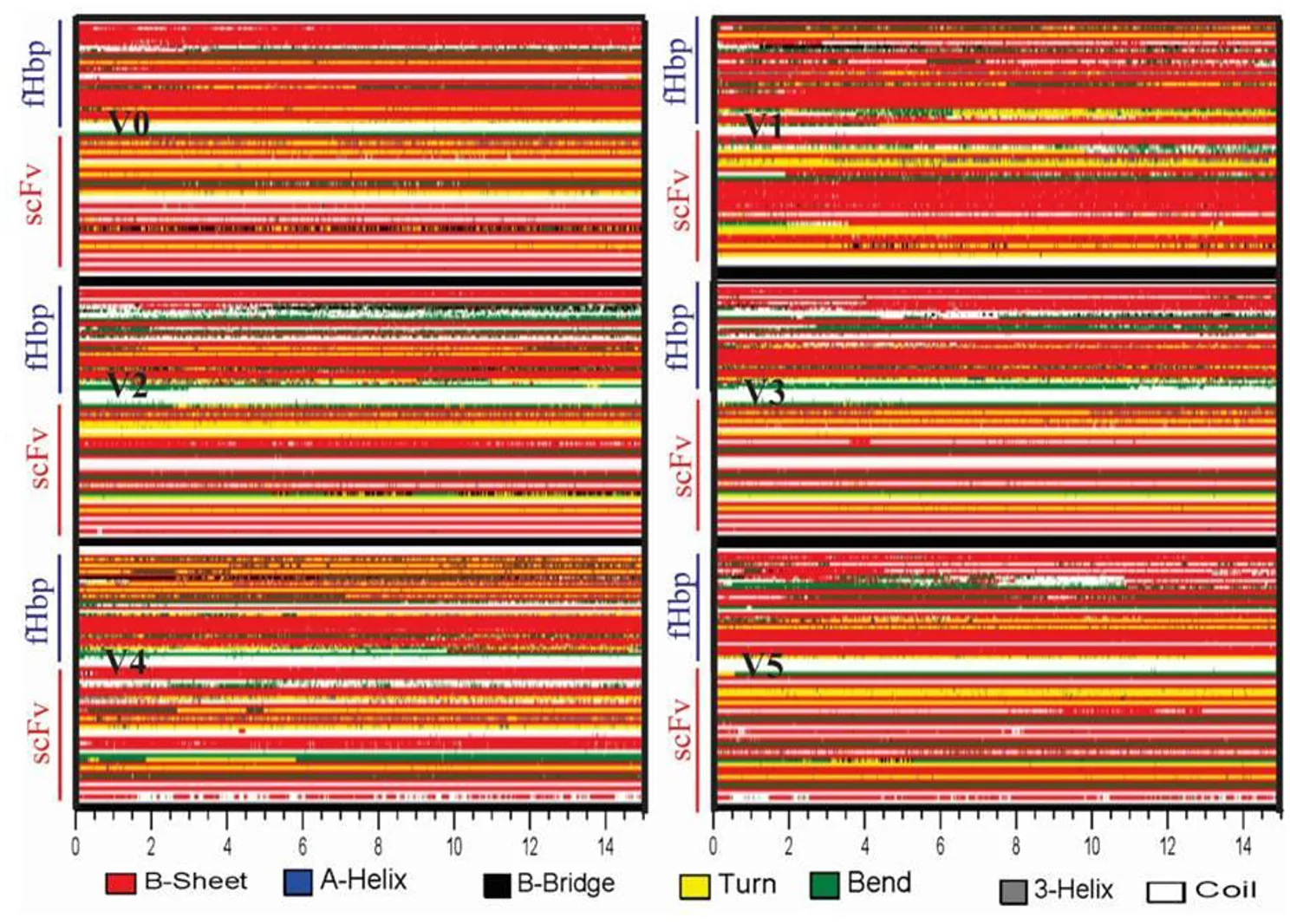
Secondary structure evolution of fHbp, scFv (native and V3 (R99D) variants), and scFv–fHbp complexes during the 15 ns molecular dynamics simulation. The simulation timeline (0–15 ns) is shown on the x-axis, and secondary structure assignments are indicated by color coding: red (β-sheet), blue (α-helix), black (β-bridge), yellow (turn), green (bend), gray (3₁₀ helix), and white (coil). Comparative panels display the native antibody fragment and its engineered variants, including V0, V1, V2, V3, V4, and V5, allowing visualization of secondary structure stability and transitions throughout the simulation. Among the variants, V3 demonstrates relatively consistent preservation of ordered structural elements, suggesting improved conformational stability within the complex compared with the other variants

DSSP analysis was performed on all protein entries in the PDB to identify secondary structural changes in the variants, because secondary structure is critical for protein function and its disruption can lead to structural irregularities. Such variations often involve interconversion between turns and random coils. Owing to its high mobility, the N-terminus typically shows the greatest secondary structure variability. Figure 3 demonstrates that the designed variants exhibited no significant secondary structural changes, further supporting their stability.

### Hydrogen bond patterns

**Fig. 4:**
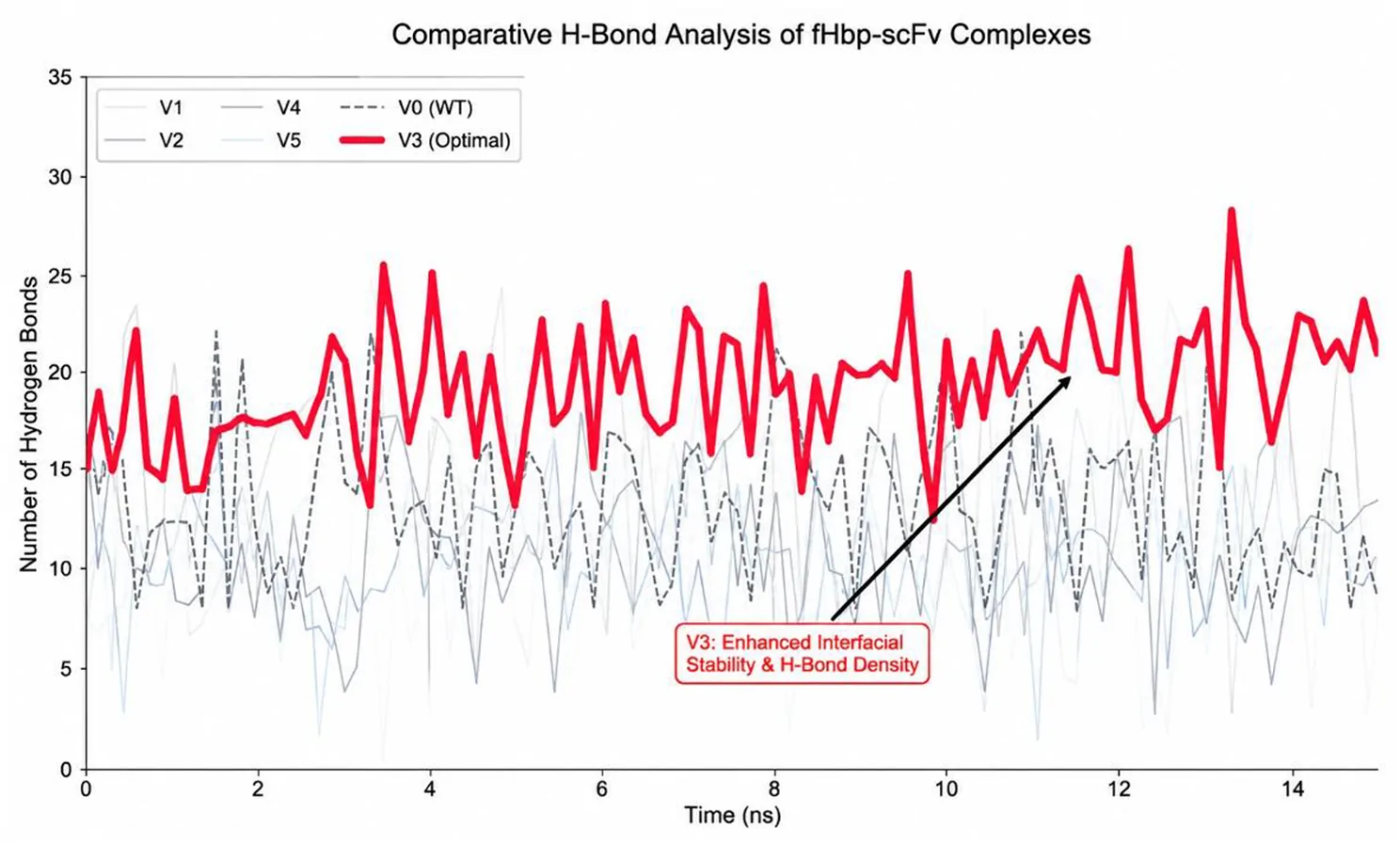
Number of inter- and intramolecular hydrogen bonds in the simulated complexes. The number of hydrogen bonds formed at the protein–protein interface was monitored over a 15 ns molecular dynamics trajectory. The V3 variant (highlighted in bold red) exhibits a consistently higher and more stable hydrogen-bonding network than the wild-type (V0) and the other engineered variants (V1, V2, V4, and V5, represented in shaded gray). This enhanced hydrogen-bond density in V3 suggests superior interfacial complementarity and stronger binding affinity, contributing to the overall stability of the complex

Intermolecular hydrogen bonds are among the most important interactions stabilizing secondary structural elements, namely α-helices and β-sheets, and the number of these bonds can substantially influence ligand–receptor binding affinity. Figure 4 shows the number of inter- and intramolecular hydrogen bonds for each simulation. As shown in Fig. 4, the number of hydrogen bonds in the scFv, fHbp, and variant scFv–fHbp complexes exhibited only minor fluctuations.

The number of intramolecular hydrogen bonds within the scFv–fHbp system increased first in V1 and subsequently in V3, suggesting that the greater number of intramolecular hydrogen bonds in these variants may be associated with stronger binding affinity to fHbp than in the other variants.

**Fig. 5:**
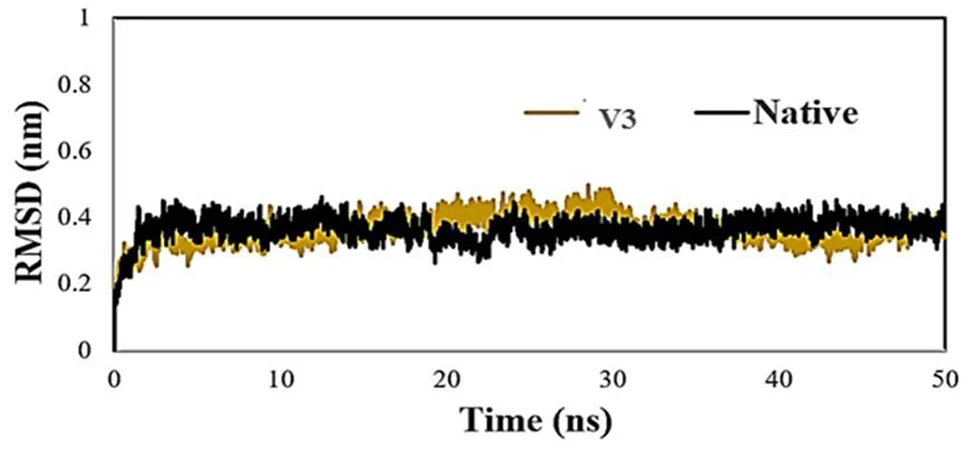
RMSD trajectories of the scFv–fHbp complex over 50 ns of all-atom molecular dynamics simulation. RMSD values (in nm) were calculated for backbone atoms relative to the initial equilibrated structure. The plot compares: Native complex (V₀, black line) V₃ mutant complex (R99D substitution, brown line)

**Fig. 6:**
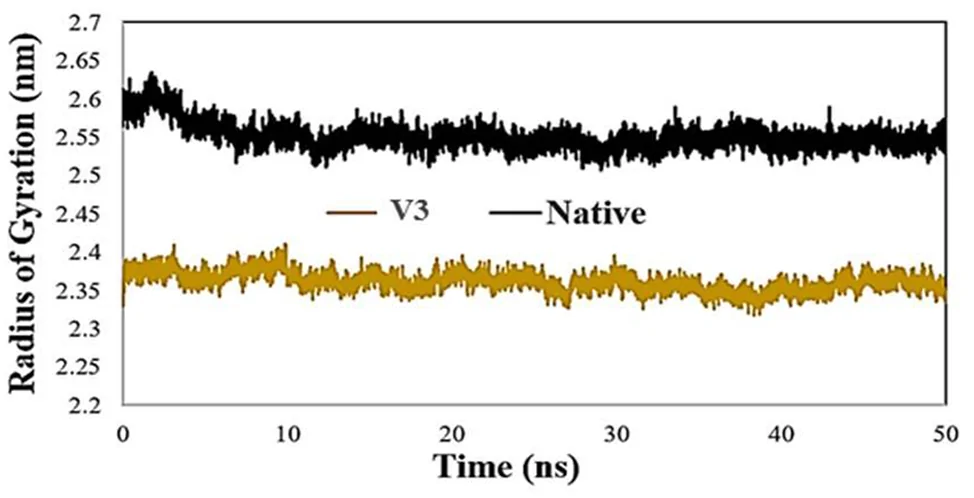
Radius of gyration (Rg) of the native scFv–fHbp complex (V0; black line) and V3 (R99D) (brown line) during 50 ns of all-atom molecular dynamics simulation

**Fig. 7:**
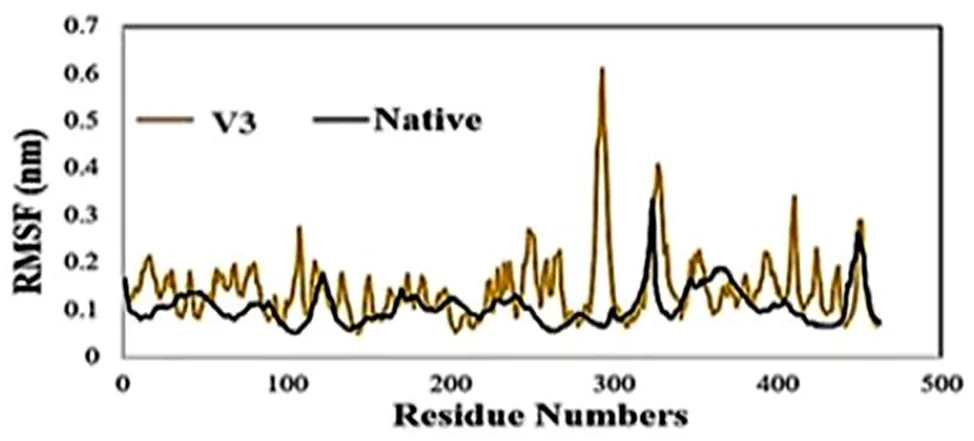
Per-residue RMSF (Cα atoms, in nm) for the scFv–fHbp complex over the 50 ns MD simulation: native (V₀, black line) versus V₃ mutant (R99D, brown line). Both profiles show low fluctuations (~ 0.1–0.2 nm) in structured core regions

Considering MD-derived parameters such as RMSD and radius of gyration (Rg), V3 demonstrated the best performance among the selected variants during the 15 ns simulation. Consequently, the V3 (R99D) mutant scFv–fHbp complex was reanalyzed using MD metrics, including RMSD, Rg, and RMSF, over a 50 ns simulation for further validation. Figures 5 and 6, and 7 present the results of these parameters, respectively.

As shown in Fig. 5, both trajectories exhibit low and stable RMSD values (approximately 0.3–0.5 nm after the initial equilibration), indicating excellent preservation of the overall complex structure and interface integrity throughout the extended 50 ns simulation. In addition, RMSF values for the native and V3 variants were derived from the simulations. RMSF reflects residue-level mobility throughout the 50 ns trajectory. Figure 7 shows distinct flexibility patterns for the native scFv and the V3 mutant scFv in complex with fHbp. The residues involved in the V3–fHbp interaction exhibit slightly reduced flexibility (lower RMSF) than those in the native complex, suggesting that these residues are more rigid in the V3–fHbp complex.

Figure 6 shows that both Rg profiles remained highly stable throughout the extended simulation, with the native complex maintaining an Rg of approximately 2.55–2.60 nm and the V3 mutant consistently showing lower values of approximately 2.30–2.35 nm.

**Fig. 8:**
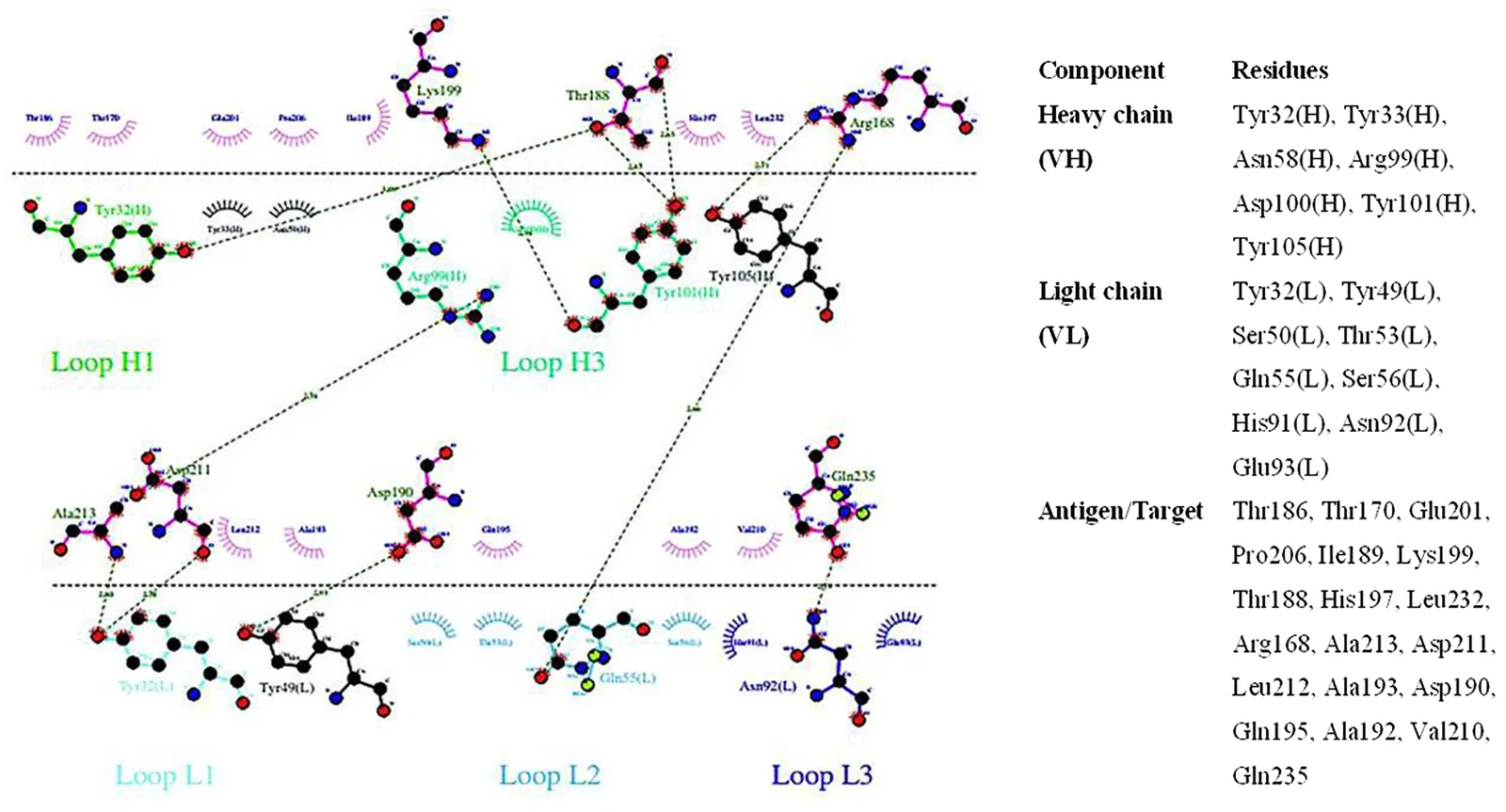
Two-dimensional LIGPLOT representation of the antibody–antigen interface in the native scFv–fHbp complex. Heavy-chain CDR loops H1 and H3, together with light-chain CDR loops L1–L3, interact with fHbp through hydrogen bonds (green dashed lines) and hydrophobic contacts (red arcs), illustrating the network of polar and non-polar interactions that stabilize the complex

**Fig. 9:**
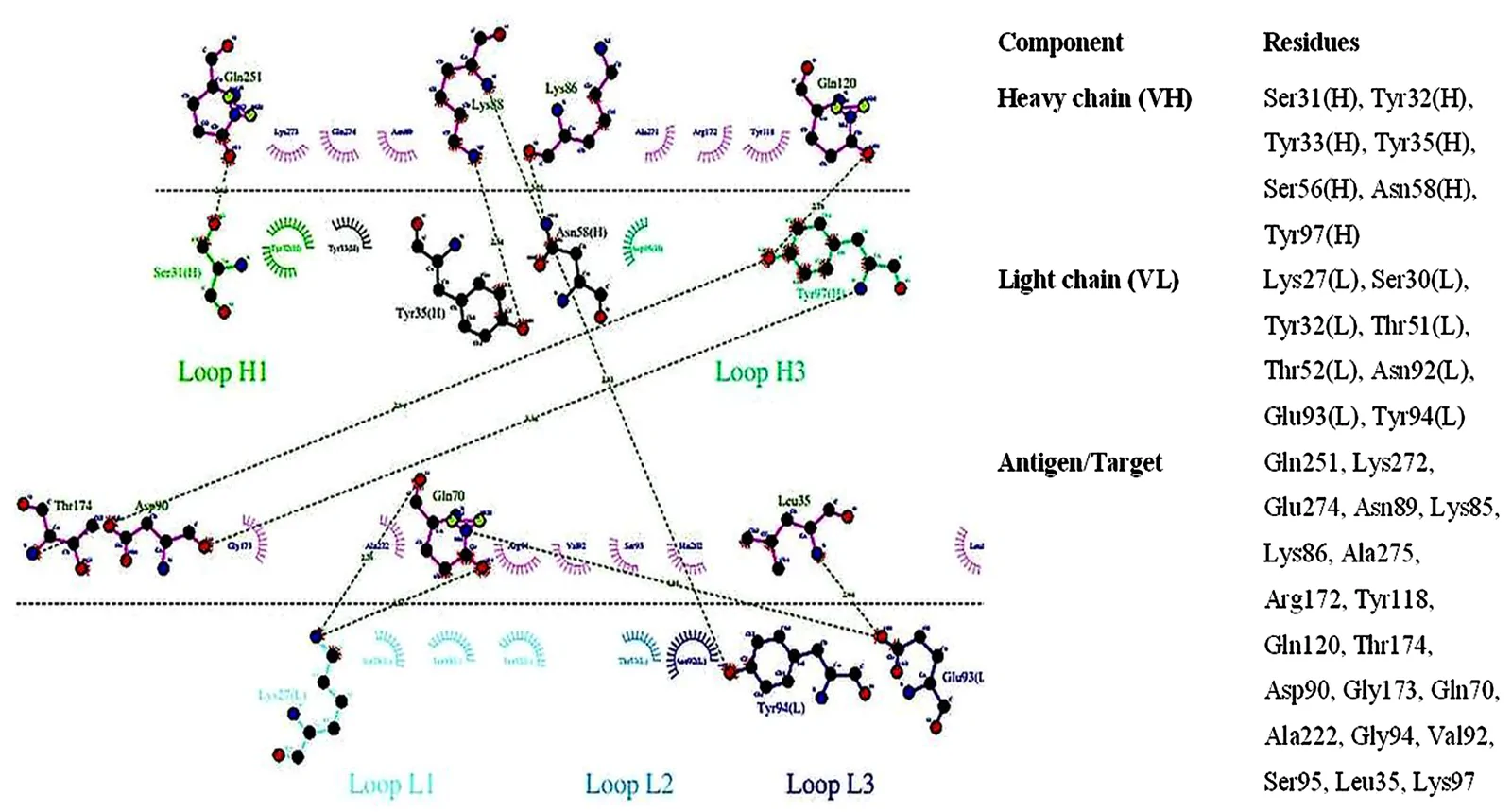
Two-dimensional LIGPLOT representation of the scFv–fHbp (R99D) complex. The interface is mediated by heavy-chain CDRs H1 and H3 and light-chain CDRs L1–L3, with hydrogen bonds shown as green dashed lines and hydrophobic/nonbonded contacts indicated by arc symbols. The diagram highlights the network of interactions between antibody residues and fHbp

Residues of the scFv that participate in interactions with fHbp were identified using the LIGPLOT program (Figs. 8 and 9). Analysis of both the native (V0) and V3 (R99D) scFv–fHbp complexes with LigPlot revealed hydrogen bonds, hydrophobic contacts, and corresponding bond lengths involving hydrogen atoms from both the scFv and fHbp.

### Expression and purification of the scFv

**Fig. 10:**
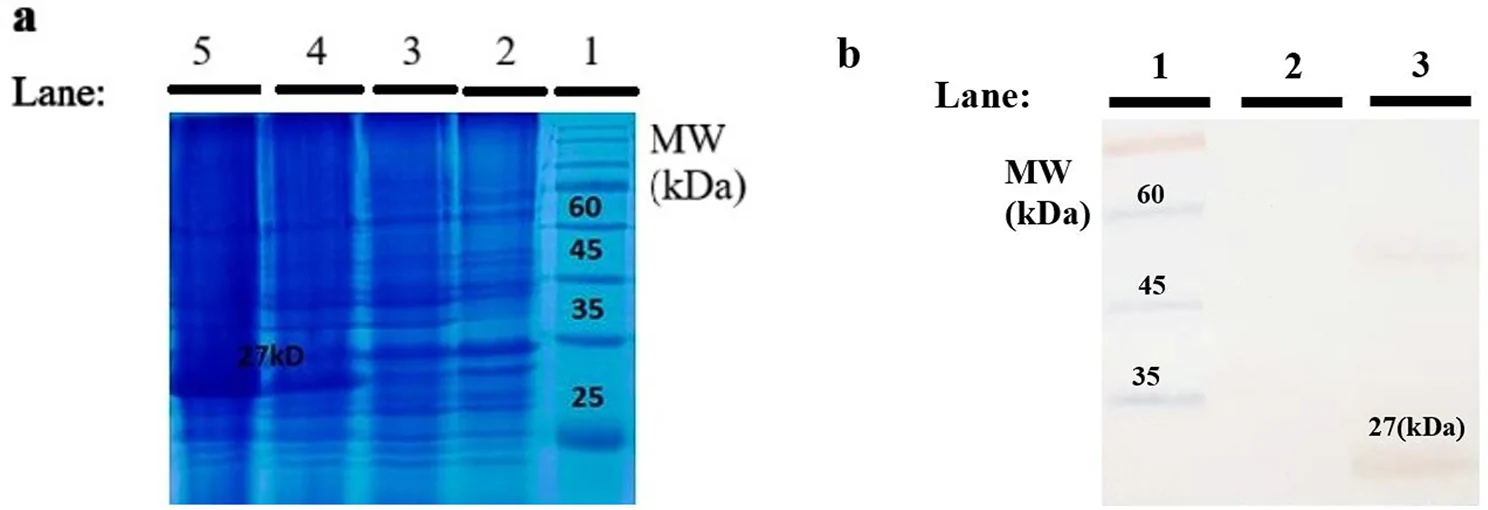
SDS-PAGE analysis of V3 (R99D) scFv protein expression at various time points and IPTG concentrations: from right to left. (**a**) Lane 1, marker; lane 2, BL21; lane 3, t0; lane 4, t2 (IPTG 1 mM); lane 5, t2 (IPTG 1 mM). Western blot analysis of V3 (R99D) scFv protein: (**b**) Lane 1, marker; lane 2, BL21; lane 3, purified protein

The V3 (R99D) mutant scFv was expressed in *Escherichia coli* BL21(DE3) and purified by nickel-NTA agarose affinity chromatography. SDS-PAGE and Western blot analyses of the purified scFv (27 kDa) are shown in Fig. 10. The scFv concentration was 0.40 mg/mL.

### Characterization of purified scFv by indirect ELISA

**Fig. 11:**
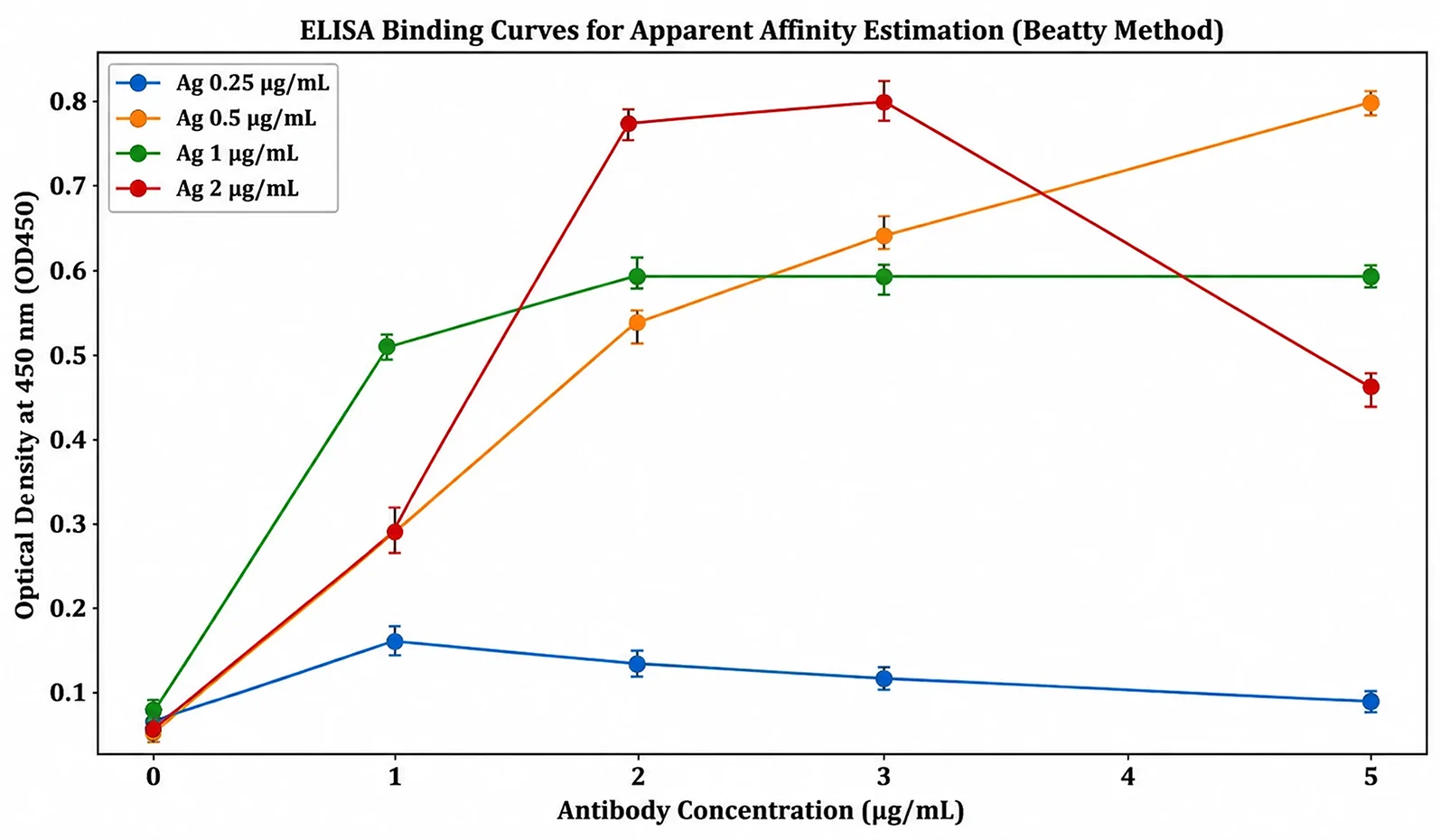
Determination of scFv binding to fHbp by indirect ELISA using the Beatty method. The binding affinity of the recombinant scFv antibody toward factor H-binding protein (fHbp) was evaluated by indirect ELISA using two antigen coating concentrations according to the Beatty method. Microtiter plates were coated with fHbp at four different concentrations and incubated with serial dilutions of purified scFv. Bound scFv was detected using an HRP-conjugated secondary antibody, and absorbance was measured at 450 nm (OD₄₅₀). Data points represent the mean ± SD of three independent biological replicates. Binding curves were generated from the measured absorbance values to estimate the relative affinity of the scFv–fHbp interaction. Data points represent the mean of replicate measurements, and error bars indicate the standard deviation (SD)

To estimate the binding affinity of the scFv, ELISA binding curves were generated at various fHbp coating concentrations (Fig. 11). The binding profiles exhibited concentration-dependent saturation, with data points representing the mean OD₄₅₀ ± SD of triplicate measurements. The linear portions of these curves were used to calculate the apparent K_aff using the Beatty method, providing a robust estimate of scFv–fHbp interaction strength.

Affinity analysis revealed an affinity constant of 67.93 ± 0.02 × 10⁹ M⁻¹ (mean ± SD, *n* = 3 biological replicates) for the purified scFv. Accordingly, this V3 (R99D) scFv exhibited a higher affinity for the recombinant fHbp protein than the native form of the scFv [9].

## Discussion

The findings of the present study demonstrate the effectiveness of an integrated computational-experimental strategy for substantially enhancing the antigen-binding capacity of a single-chain variable fragment (scFv) directed against factor H-binding protein (fHbp), a critical surface-exposed virulence determinant that enables *Neisseria meningitidis* to evade host complement-mediated immunity. This approach is consistent with prior reports in which in silico-guided affinity maturation has proved instrumental in optimizing antibody-like binders. For example, Ebrahimi et al. used homology modeling and molecular dynamics to redesign nanobodies targeting the PLGF receptor; they identified key interfacial residues, generated mutants, docked them to select low-energy poses, and ultimately validated four high-affinity candidates through simulation [18]. Similarly, Ghaffari et al. rationally engineered affibody variants against HER2 by exploiting electrostatic complementarity, with MD simulations confirming superior receptor engagement for one charge-introduced mutant over another [19]. Payandeh et al. applied docking and dynamics to mutate ofatumumab residues involved in CD20 binding, yielding variants with markedly improved predicted affinity [20]. Comparable workflows have also been used to refine bevacizumab for VEGF binding in ovarian cancer models [21] and to generate stabilized scFv libraries against dengue envelope epitopes [22]. More recently, such principles have been extended to scFv mutants against small molecules such as amantadine, producing up to a 3.9-fold increase in sensitivity through targeted Gly-to-Phe substitution at a key site [23].

In the present investigation, the parental scFv model was reliably constructed using PIGSpro (http://biocomputing.it/pigspro), which leverages an expanded template repository together with refined loop and orientation optimization to produce biophysically interpretable structures that remain competitive with modern deep-learning approaches [10, 12]. Subsequent ClusPro docking [13] delineated critical interface contacts, thereby enabling the semi-rational design of approximately 300 point mutants.

Binding-energy evaluation provided additional insight into affinity improvement. The observed reduction in binding energy for the optimized variant reflects strengthening of key intermolecular interactions and is consistent with the enhanced complex stability observed in our MD simulations. Notably, recent computational frameworks, such as those discussed by Biswas et al. [24], underscore the value of accurate binding-energy estimation as a reliable predictor of protein-protein interaction stability and functional integrity. By aligning our analyses with such methodologies, we confirmed that the selected point substitution effectively anchored the scFv within the fHbp binding pocket, thereby accounting for the observed increase in affinity.

Rigorous filtering through all-atom MD simulations ultimately identified the R99D substitution in VH framework region 3 as the optimal variant. This arginine-to-aspartate exchange, positioned adjacent to CDR-H3, introduced a localized charge reversal that conferred exceptional conformational stability, as evidenced by minimal RMSD and Rg fluctuations, reduced per-residue flexibility (RMSF) at interface residues, and sustained intramolecular hydrogen-bonding throughout the extended trajectories. Comparative LIGPLOT analyses revealed reorganized polar and hydrophobic interaction networks that enhanced electrostatic steering and interfacial complementarity without perturbing the global fold architecture [25, 26].

Experimental validation further corroborated the computational findings. The R99D variant yielded an ELISA-determined affinity constant (K_aff) of 67.93 × 10⁹ M⁻¹, representing a marked improvement over the native scFv (approximately 8.65 × 10⁹ M⁻¹) [9]. It should be emphasized that docking scores, MD-derived stability descriptors, and experimentally measured affinity are related but not identical metrics. In the present study, ClusPro docking was used primarily as an initial screening tool to identify favorable interface geometry and electrostatic complementarity among candidate variants. Accordingly, docking scores were interpreted as qualitative ranking criteria rather than direct quantitative predictors of binding affinity. Likewise, MD-derived parameters such as RMSD, Rg, RMSF, DSSP, and hydrogen-bond profiles were used to assess structural stability, compactness, and preservation of the binding interface during simulation, but these metrics do not directly measure binding free energy or equilibrium affinity constants. Within this framework, the R99D variant showed consistent behavior across computational and experimental analyses: it ranked favorably in docking-based screening, exhibited lower structural fluctuations and a more stable interface during MD simulations, and ultimately demonstrated improved apparent affinity in the ELISA assay. Nevertheless, this agreement should be interpreted qualitatively rather than as a strict quantitative correlation between docking score, MD stability metrics, and experimental affinity. Docking is limited by simplified scoring functions and incomplete treatment of conformational flexibility, solvation, and entropy, whereas MD stability descriptors reflect dynamic behavior rather than thermodynamic binding strength. In addition, the ELISA-based value reported here represents an apparent affinity measurement for the selected variant rather than a full thermodynamic comparison of all designed mutants.

Although the GROMOS96 43a1 force field, combined with the SPC water model, adequately described short- to intermediate-timescale interface behavior in this system, future studies may benefit from more contemporary parameter sets, such as CHARMM36m or AMBER ff19SB, coupled with enhanced-sampling protocols, such as Gaussian accelerated MD, to better capture infrequent conformational states and entropic contributions.

Likewise, although ClusPro enabled efficient rigid-body sampling, integration with state-of-the-art AI-augmented docking pipelines or AlphaFold3-generated conformational ensembles could further improve pose discrimination and epitope specificity in subsequent optimization rounds. Overall, this work delineates a streamlined and mechanistically coherent pathway for scFv affinity enhancement, one that seamlessly bridges homology-based modeling, physics-based virtual screening, and empirical validation, and thus offers a compelling framework for accelerating the development of potent antibody fragments against high-priority bacterial pathogens.

Although the present study primarily relied on molecular dynamics-based stability descriptors to assess model robustness, additional model-quality validation using Ramachandran plot analysis, ERRAT, ProSA, or Verify3D would provide further confidence in the structural reliability of the designed variants and should be considered in future work. Future studies may also benefit from complementary biophysical validation, such as isothermal titration calorimetry or related calorimetric approaches, to quantify the thermodynamic parameters underlying the scFv–fHbp interaction.

## Conclusion

This study combined in silico and experimental approaches to improve the affinity of an scFv antibody for fHbp. Residues Tyr35, Leu31, Arg99, and Ala100 were identified as key contributors to the interaction. The Arg99Asp mutation produced the greatest improvement in binding score, with no significant changes observed in the MD analysis. These results suggest that ligand-based affinity enhancement is a promising strategy for designing high-affinity antibody fragments.

## Supplementary Information

Below is the link to the electronic supplementary material.

Supplementary Material 1

Supplementary Material 2

Supplementary Material 3

Supplementary Material 4

## Data Availability

The datasets generated during and/or analyzed during the current study are not publicly available but are available from the corresponding author on reasonable request.
