# Supplementary material for: Affinity improvement of specific scFvs interacting with the Neisseria meningitidis factor H-binding protein: a molecular dynamics simulation approach and experimental study
Source: BMC Biotechnol. 2026 Jul 25;26:122. doi: 10.1186/s12896-026-01204-3 (PMC13685201; doi:10.1186/s12896-026-01204-3)
Supplement: Supplementary file 2 [file 12896_2026_1204_MOESM2_ESM.docx]

**Supplementary Table S1**

**Summary of the mutation-design workflow, screening criteria, and variant prioritization.**

| **Stage** | **Process /**  **Step** | **Description / Criteria** | **Outcome** |
| --- | --- | --- | --- |
| **Stage1** | Initial design | Design of approximately 300 single-  point mutants targeting residues at the  scFv-fHbp interaction interface. | Initial mutant  library generated |

Candidates were evaluated based on

| **Stage2** | Screening | spatial proximity to the interface, | Reduced pool of |
| --- | --- | --- | --- |
|  |  | hydrogen-bonding potential, |  |
|  |  |  | candidates |
|  |  | electrostatic complementarity, and |  |

steric compatibility.

| **Stage3** | Prioritization | Top candidates were further assessed | Five variants were |
| --- | --- | --- | --- |
|  |  |  | selected: Y35H, |
|  |  | by molecular dynamics simulation and |  |
|  |  |  | L31R, R99D, R99H, |
|  |  | stability analysis. |  |
|  |  |  | and A100K |

**Note:** This table summarizes the rational and semi-rational workflow used to reduce the initial set of approximately 300 single-point mutants to the five variants selected for detailed analysis and experimental validation.
