# Supplementary material for: Affinity improvement of specific scFvs interacting with the Neisseria meningitidis factor H-binding protein: a molecular dynamics simulation approach and experimental study
Source: BMC Biotechnol. 2026 Jul 25;26:122. doi: 10.1186/s12896-026-01204-3 (PMC13685201; doi:10.1186/s12896-026-01204-3)
Supplement: Supplementary file 3 [file 12896_2026_1204_MOESM3_ESM.docx]

Dear editor:

I posted the 12% gel image to the manuscript, because the 10% SDS-PAGE gel lacked a marker. I will replace the 12% gel if appropriate.
