# Supplementary material for: Affinity improvement of specific scFvs interacting with the Neisseria meningitidis factor H-binding protein: a molecular dynamics simulation approach and experimental study
Source: BMC Biotechnol. 2026 Jul 25;26:122. doi: 10.1186/s12896-026-01204-3 (PMC13685201; doi:10.1186/s12896-026-01204-3)
Supplement: Supplementary file 4 [file 12896_2026_1204_MOESM4_ESM.docx]

**Supplementary File S1. Amino acid sequence of the V3 (R99D) scFv**

**The amino acid sequence of the V3 (R99D scFv is provided below:**

QVHLEQSGAQLVKPGASVKLSCKASGYTFTLYYMYWVKQRPGQGLEWIGEINPSNGGTNFNEKFKSKATLTVDKSSSTAYMQLSSLTSEDSAVYYSAMDAYAMDYWGQGTTVTVSSGGGGSGGGGSGGGGSDIELTQSPSYLAASPGETITINCRASKSISKYLAWYQEKPGKTNKLLIYSGSTLQSGIPSRFSGSGSGTDFTLTISSLEPEDFAMYYCQQHNEYPLTFGAGTKLELKRADAA

**Note:** The R99D substitution was introduced in the heavy chain to improve affinity.
